# New Insights into the Variation and Admixture of the Cave-Dwelling Spider *Trogloneta yunnanensis* in South China Karst

**DOI:** 10.3390/ani13071244

**Published:** 2023-04-03

**Authors:** Shiliang Liu, Chuang Zhou, Yucheng Lin

**Affiliations:** 1 Key Laboratory of Bio-Resources and Eco-Environment (Ministry of Education), College of Life Sciences, Sichuan University, Chengdu 610064, China; 2 The Sichuan Key Laboratory for Conservation Biology of Endangered Wildlife, Sichuan University, Chengdu 610064, China

**Keywords:** *Trogloneta yunnanensis*, South China Karst, population genetics, diversity and divergence, phylogenetic analysis

## Abstract

**Simple Summary:**

The subterranean fauna is an important component of global biodiversity. However, research on the subterranean fauna of the Yunnan–Guizhou Plateau in Southwest China, one of three important karst landforms in the world, is limited. In this study, we performed a population genetic analysis and reconstructed the phylogenetic tree of six populations of *Trogloneta yunnanensis* in South China Karst. The results showed that there was high genetic divergence among six populations, and the divergence of these six populations can be traced back to the late Pleistocene. Our results suggested that isolation was a pivotal factor affecting the biodiversity of cave faunas, and the biodiversity of cave-dwelling faunas needs to be studied as soon as possible.

**Abstract:**

Subterranean karst caves can contain unexpected biodiversity, but few studies related to spider population genetics have been conducted in the karst area of Southern China. In this study, we investigated the population genetic structure of *Trogloneta yunnanensis* (Song & Zhu, 1994) based on 73 spider samples from six underground populations in South China Karst. Population genetic structure analysis showed a clear divergence (*F*_ST_ > 0.9 and Nm < 0.05) among populations according to mitochondrial genes. The phylogenetic gene tree constructed by BI and ML methods recovered six geographic clades. Divergence time estimation indicated that the divergence of these six populations can be traced back to the late Pleistocene. We supposed that the geographic isolation led to the extreme population structure. According to this study and previous studies about troglobites living in this region, the subterranean habitats of the Yunnan-Guizhou Plateau may contain many organisms with similar genetic structures. The subterranean biodiversity in the karst area of Southern China needs to be re-evaluated and protected.

## 1. Introduction

Biodiversity loss is one of the most serious environmental crises worldwide; therefore, it is important and urgent to study it [1]. Compared with surface species, subterranean fauna is less studied due to sampling difficulty, low population density, and the rarity of encountering some species [2]. Caves provide a unique habitat for organisms, where there is no or less sunlight, no or less plant growth, high CO_2_ concentration, constant temperature close to the mean annual region temperature, and scarcity of food [3]. Culver and Holsinger [4] estimated a global total of 50,000 to 100,000 obligate subterranean species. Because of the extreme environment, cave-adapted species tend to have a relatively simple structure of a community and are isolated from each other in time and space [5]. Thus, the cave faunas can help us understand the evolution and biogeography of species and speciation under geographic isolation [6,7,8]. The contribution of endemic and relict taxa to overall biodiversity are increasingly being reappraised for many habitats and living groups from the conservation perspective [9].

Many cave species have extremely small ranges, with a large part of troglobitic species, and subspecies limited to a single county, and many species are even known from a single cave [10]. Isolation barriers between cave systems limit gene flow between populations of cave organisms, promoting differentiation among populations and effectively dividing parts of the cave systems into subterranean islands [11]. Many studies in Europe and North America have found strong genetic differentiation among populations of cave faunas, particularly cave terrestrial invertebrates [5,11,12,13,14,15]. However, some studies indicated that cave species also have moderate to high rates of gene flow [12,16]. The gene flow may attribute to the existence of subterranean interconnecting passages or recent secondary contact between subterranean and surface forms [16]. The diversity of cave-dwelling species was influenced by many factors, such as the intrinsic characteristics of each species, the degree of cave dependence of species, and the distribution of limestone in karst areas [6,16,17,18]. The role of caves as natural laboratories has not been fully explored, especially when macroecological and biogeographic patterns at a continental or global scale are considered [19].

The Mountains of Southwest China are one of the 34 global biodiversity hotspots [20]. The Yunnan-Guizhou Plateau in Southwest China is one of three important karst landforms in the world [21], where thousands of caves exist. There are some studies related to cave fish in this area [22,23,24], but terrestrial cave invertebrates which are regarded to make up the majority of subterranean faunas are still understudied [25]. The biodiversity of cave-dwelling faunas needs to be studied as soon as possible, which are threatened by a range of climate change and human activity [26].

In this study, we investigated the population genetics of a tiny cave spider, *Trogloneta yunnanensis* [27], which belonged to the family Mysmenidae Petrunkevitch, 1928. To date, this species was only found in underground caves of the Yunnan-Guizhou Plateau, usually living under humid rocks or in rock gaps [28]. *Trogloneta yunnanensis* has been showing some cave-adapting characteristics, such as lighter color skin with little pigmentation. The main aims of this study were: (1) to explore the genetic structure of *Trogloeta yunnanensis* populations; (2) to reconstruct phylogenetic relationships and estimate the divergence time of different populations.

## 2. Materials and Methods

### 2.1. Sampling

**Figure 1:**
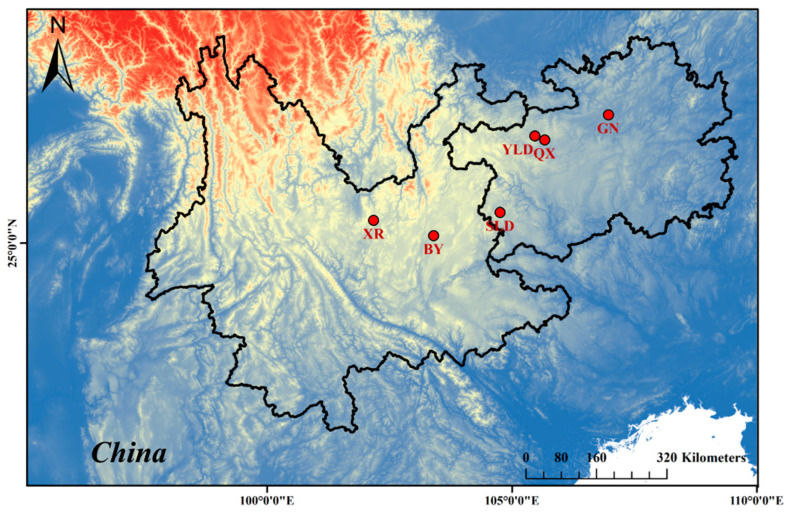
Sampling cave locations indicated by red dots.

The samples of spiders were collected from six isolated caves in Southwestern China (Figure 1). The sampling locality information is provided in Table 1. Sampled individuals were preserved in 95% ethanol in the field and then stored at −20 °C in the key Laboratory of Bio-resources and Eco-environment after being taken back. We identified spider species by the morphology of copulatory organs in both sexes. In this study, we found no surface populations of *T. yunnanensis* outside the cave, and there were no surface records of *T. yunnanensis* individuals in previous studies [28,29].

### 2.2. DNA Extraction, Sample Preparation, and Gene Sequencing

Depending on the abundance of specimens, ten to fourteen individuals per cave were selected to extract DNA. *Trogloneta yuensis* [30] *Yamaneta kehen* (MK895531, MK908789, MK908805, MK908797, MK895538) and *Yamaneta paquini* (MK895536, MK908794, MK908810, MK908802, MK895544) were selected as outgroups. *Trogloneta yuensis* is the most closely related species to *T. yunnanensis*, and two *Yamaneta* species are also cave-dwelling mysmenid spiders from the Mountains of Southwest China [31]. We used DNeasy Blood and Tissue Kit (Qiagen, Hilden, Germany; P/N: 69506) to extract genomic DNA from the prosomal tissue of 73 individuals according to the instructions (the abdomens and male palps were kept as vouchers). We sequenced two partial mitochondrial genes and three partial nuclear genes: cytochrome c oxidase subunit 1 (*cox1*), *16S* ribosomal RNA (rRNA), *28S* rRNA, *H3,* and *ITS-2*. The primers were provided in Table S1. Two × M5 HiPer plus Taq HiFi PCR mix with blue dye was used as the polymerase enzyme. PCR reactions were 30 s at 94 °C, 30 s at 45 °C to 55 °C, and 30 s at 72 °C (×35 times). PCR products were sent to the Chengdu Branch of Qingke Biotechnology Co., LTD for sequencing. The sequencing data were checked and edited using Bioedit 7.2.5 [32]. MEGA X [33] was used to translate and align the protein-coding sequences. Other sequences were aligned in Clustal X [34].

### 2.3. Population Genetic Analysis

We used five genes to analyze the genetic structure of six populations of *T. yunnanesis*. DNAsp v6.0 [35] was used to determine haplotypes, DNA sequence polymorphisms, the number of haplotypes (h), haplotype diversity (Hd), and nucleotide diversity (π). The TCS (Templeton-Crandall-Sing) Networks [36] were constructed in PopArt 1.7 [37]. The F-statistics and AMOVA were calculated among the populations by Arlequin 3.5 [38], and we calculated the Nm values based on the F-statistics. The uncorrected ‘p’ distances between populations were calculated by MEGA X [33].

### 2.4. Phylogenetic Analysis

To examine the monophyly and allow us to compare diversity between populations, we used the Bayesian Inference (BI) method to reconstruct the phylogenetic tree of *T. yunnanensis* based on the concatenated genes (*cox1* + *16S* + *H3* + *28S* + *ITS-2*). We used PartitionFinder2 [39] to identify the best-fit models of molecular evolution and partitioning schemes for the dataset (Table S3). The BI phylogenetic tree was constructed in Mrbayes [40], and four Markov Chain Monte Carlo (MCMCs) with default heating parameters were performed for 10,000,000 generations until the average standard deviation of split frequencies was less than 0.01. The Markov chains were sampled every 1000 generations, and the first 25% of sampled trees were burn-in.

The maximum-likelihood phylogenetic analysis was conducted in IQ-TREE v1.6.12 [41]. We used ultrafast bootstrapping with 5000 replicates [42] and the Shimodaira-Hasegawa approximate likelihood-ratio test (SH-aLRT) with 1000 replicates [43] to estimate the node support. The best-fit models were selected using ModelFinder [44]. The results are shown in Table S3.

### 2.5. Divergence Time Estimation

To estimate the divergence time among six populations of *T. yunnanensis*, the species tree was constructed in BEAST v1.10.4 [45] under the Yule process tree model. MCMC chains were run for 10 million generations, sampling every 1000 generations. All other parameters were default settings. We assessed convergence, posterior trace plots, and effective sample sizes (ESS > 200) in Tracer v.1.7.1 [46]. TreeAnnotator was used to generate a maximum clade credibility (mcc) tree with the first 25% as burn-in. The best-fit model was found in PartitionFinder2 (Table S3).

Due to the lack of suitable calibration points, we used prior information on substitution rates of genes to estimate population divergence time based on available information for spiders [13,47,48,49]. To reduce errors, we used only the *cox1* gene for divergence time estimation. Preliminary analyses using a lognormal relaxed clock for the *cox1* gene showed that the posterior distribution of the ucld.mean parameter accreted to zero, and hence a strict clock was preferred. The prior rate parameter was set to normal distribution with mean ± SD = 0.0168 ± 0.0018 for *cox1*.

## 3. Results

### 3.1. Genetic Diversity and Structure of Trogloneta yunnanensis

A total of 632 sequences were obtained from 5 gene segments (630 bp *cox1*, 420 bp *16S*, 781 bp *28S*, 312 bp *H3*, and 373 bp *ITS-2*) in 73 individuals of *T. yunnanensis*. However, several *cox1*, *28S*, and *ITS-2* sequences could not be recovered because of their high AT content. The details are provided in Table S2. Since these sequences were similar among populations of *T. yunnanensis*, we believe that these missing data did not affect our analyses. The *cox1*, *16S*, *H3*, and *ITS-2* datasets had 30, 10, 4, and 2 polymorphic sites, respectively. The genetic diversity parameters of these six populations are summarized in Table 2. Among these six populations, QX, SLD, and YLD populations showed genetic differences in mitochondrial genes and nuclear genes, and the BY population showed high genetic diversity in mitochondrial genes (Table 2). XR and GN populations had relatively lower genetic diversity, and they only showed genetic differences in *16S* and *H3* genes, respectively (Table 2). There were no genetic differences in the *28S* gene dataset of all samples, so it was not analyzed nor discussed below.

Based on the *cox1* gene, the genetic differentiation among six populations was high, the pairwise *F*_ST_ values among populations were above 0.90, and the Nm < 0.05 (Table 3 and Table 4). Based on *F*_ST_ values, the lowest level of divergence was observed between the SLD and BY populations, and the GN and XR populations showed the highest level of divergence. The divergence between the geographically close population YLD from the QX was higher than those from the geographically much higher populations QX, BY, and SLD (Figure 1). The mean pairwise uncorrected *p*-distances of *T. yunnanensis* based on the *cox1* gene ranged from 0.49% to 2.53% (Table 5). XR and SLD were the two populations with the largest genetic distance (2.53%), while YLD and GN exhibited the smallest genetic distance (0.49%). The overall mean distance was 1.59% ± 0.31%. We also calculated the overall mean distance of 16S, H3, and *ITS-2* to be 0.73% ± 0.24%, 0.29% ± 0.18%, and 0.14% ± 0.13%, respectively.

The results of AMOVA suggested a high degree of genetic divergence among populations. A lower proportion of the variance (2.6%) was attributable to interpopulation (within populations), and nearly 97.4% of the *cox1* gene diversity was explained by variance among the different cave populations (Table 6).

**Figure 2:**
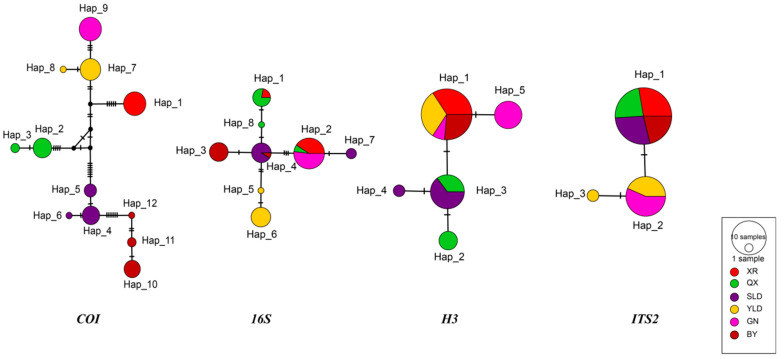
TCS network of *Trogloneta yunnanensis*. The alphanumeric code in the networks refer to haplotypes. Unsampled haplotypes are represented by small black circles.

The analysis of haplotype networks displayed clear genetic structure in populations of *T. yunnanensis*. The analysis of the *cox1* gene showed the distinct geographic structure in *T. yunnanensis*. There were 12 haplotypes in 72 individuals from six populations, and six populations had no shared haplotypes (Figure 2). The 16S haplotype network showed several shared haplotypes among different populations: XR and QX shared Hap_1, SLD and BY shared Hap_4, and XR, QX, and GN shared Hap_2 (Figure 2). The haplotype network based on nuclear genes was divided into two parts. The H3 haplotype network showed that XR, YLD, GN, and BY shared Hap_1, and QX and SLD shared Hap_3 (Figure 2). The ITS-2 haplotype network illustrated that XR, QX, SLD, and BY shared Hap_1, and YLD and GN shared Hap_2 (Figure 2).

### 3.2. Phylogenetic Relationships

**Figure 3:**
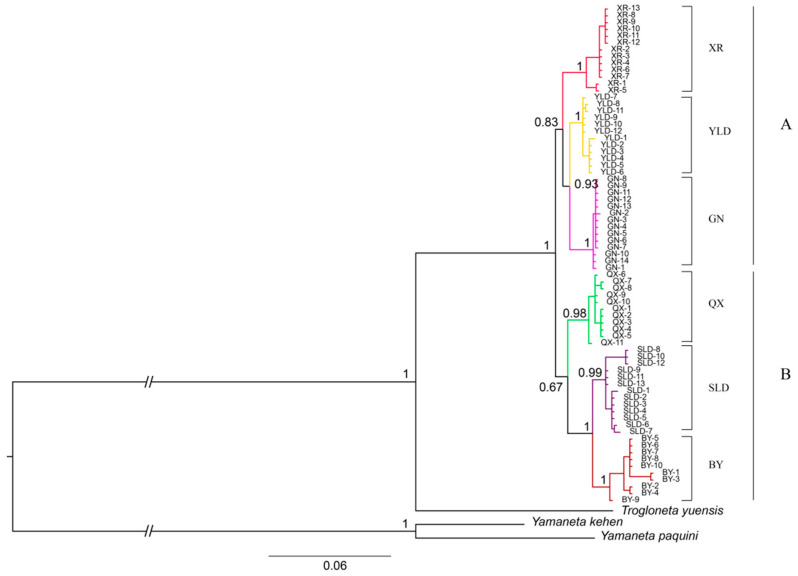
Phylogenetic tree constructed in Mrbayes inferred from concatenated genes of *Trogloneta yunnanensis*. Posterior probabilities were provided beside nodes, only given the values of major clades.

Phylogenetic analyses based on concatenated genes recovered six cave spider clades, but the topologies of the ML tree and the BI tree are inconsistent, and only given a moderate level of support values for some nodes (Figure 3 and Figure S1). The BI tree showed higher level of support values for some nodes (Figure 3). In the BI tree, *T. yunnanensis* was split into two lineages: A including XR, YLD, and GN, and B including BY, SLD, and QX. In the ML tree, YLD and GN were clustered into one lineage, and other populations (XR, SLD, QX, BY) were clustered into the second one (Figure S1), respectively. There are some “comb-like” lineages at shallow divergence levels (Figure 3), because of the same haplotype present across multiple samples of each cave.

### 3.3. Divergence Time Estimation

**Figure 4:**
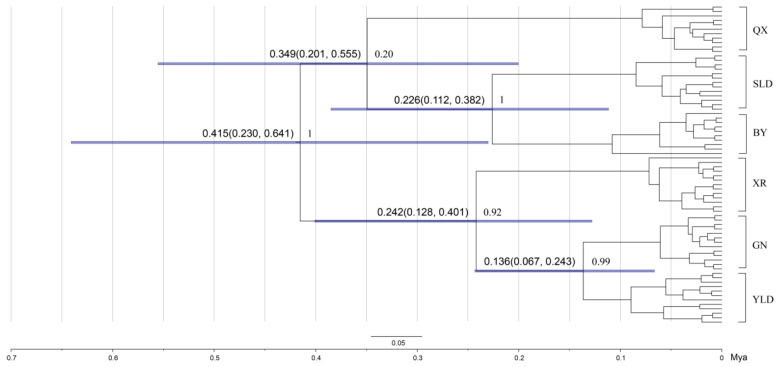
Time tree constructed in BEAST. Blue node bars indicate the 95% highest posterior density interval for divergence estimates. The divergence time (**left**) and posterior probabilities (**right**) are beside nodes. The time scale is indicated in millions of years below the tree.

The time tree was similar to the BI concatenated gene tree (Figure 4). Divergence time analysis revealed that two main clades diverged approximately 0.415 million years ago (Ma, 95% HPD = 0.230–0.641 Ma). The earliest divergent population was QX, which can be traced back to 0.349 Ma (95% HPD = 0.201–0.555 Ma), and the XR population occurred approximately 0.242 Ma (95% HPD = 0.128–0.401 Ma). The split time between BY and SLD was 0.226 Ma (95% HPD = 0.112–0.382 Ma). YLD and GN diverged into two lineages 0.136 million years ago (95% HPD = 0.067–0.243 Ma).

## 4. Discussion

### 4.1. Variation and Admixture

In this study, we did not find the existence of *T. yunnanense* outside the cave. There are no records of *T. yunnanense* epigean individuals. According to the morphological characteristics of *T. yunnanense*, we suppose that it is an obligate cave species. According to the analysis results of the *cox1* gene, the population genetic structure of *T. yunnanensis* indicated a pattern of low intra-population diversity and high inter-population diversity. The genetic variation within each population of *T. yunnanensis* was extremely small, and the number of polymorphic (segregating) sites ranged from 0 to 3. The genetic diversity varied significantly among geographical distinct populations of *T. yunnanensis.* The lack of shared haplotypes among these six populations (Figure 2) and the high *F*_ST_ values (>0.4, Table 3) indicated that there was currently little to no migration of spiders between caves. The results were similar to previous studies associated with cave spiders [11,12,13]. The pattern of genetic structure of the *T. yunnanensis* population was consistent with the predicted results of the population model in a fragmented habitat, which was mainly caused by geographical isolation and habitat. Species trapped in caves are unable to exchange genes with the outside individuals and develop further genetic differentiation [11]. Because of the special environment in caves and small population size, cave faunas may form extreme genetic structures. The genetic distance among different populations of *T. yunnanensis* ranged from 0.49% to 2.5%, and there was no significant correlation in geographical distance. The closest genetic distance is between YLD and GN of populations, but their *F*_ST_ was 0.96, which revealed a clear divergence between them.

Such large genetic differences among populations have led us to consider the possibility of the existence of cryptic species. Cryptic species have been described in many taxa, such as birds [50], reptiles [51], amphibians [52], crustaceans [53], and others. Previous studies of cave arthropods in karst areas of southern China have found that the underground fauna contained a surprising diversity. Zhang and Li [54] have found that the *Nesticella* cave spiders inhabit Yunnan–Guizhou Plateau invaded caves only recently, and cryptic species probably exist within them because of the deep divergences within the species. A study by Zhang and Li [25] on the cave telemid found that *Telema cucurbitina* was a species complex with a genetic distance of *cox1* among different cave populations ranging from 5.4% to 17.3%. They concluded that multiple cryptic species existed in the population of *Telema cucurbitina*. The genetic distance among populations of *T. yunnanensis* was much smaller than that of *Telema cucurbitina*, so we excluded the possibility that there were cryptic species in *T. yunnanensis*. Analysis of nuclear genes revealed a more conserved genetic structure, dividing the six populations into two clusters. It was possibly that maternal inheritance of mitochondrial genes reduced the effective gene flow by a factor of four compared with diploid nuclear systems [12].

### 4.2. Impact of Geographical Isolation

We reconstructed the phylogenetic tree of *T. yunnanensis* based on concatenated genes, showing the high levels of support values for main clades. The populations of *T. yunnanensis* were genetically isolated, and each population was supported as a monophyletic group. Highly different lineages (YLD and QX) were observed occupying geographically adjacent areas, but there was no mixing, suggesting that migration was limited (Figure 1 and Figure 3). The phylogenetic tree showed that BY and SLD were sister groups, which indicated that they have the closest and most stable relationship among these six populations. The topological structure of the BI tree and the ML genes tree was different, maybe because of the recent differentiation of the six populations. We believe that the dispersal ability of *T. yunnanensis* is limited, and cave isolation has a significant impact on its genetic structure.

### 4.3. Evolutionary History

The colonization and speciation of cave animals is generally explained by two hypotheses, one is the “climate-relict”, and the other is the “adaptive-shift” [3,5]. The first model was proposed for continental temperate ecosystems, where the surface species colonize to the cave environment. The isolation of cave populations occurs as surface populations become extinct (due to climatic changes). Under the second model, with the active colonization of surface populations in cave environments, adaptive differentiation occurred between populations on the surface and in the cave, and gene flow decreased. In Asia, a general cooling has gradually replaced the warm and humid climates of the early Miocene [55]. Climate change has a greater impact on vegetation cover, especially in the mid-latitudes [55]. This includes the slow but steady decline of the once widely distributed warm-temperate evergreen forests, which have gradually moved to coastal and low-latitude regions and been replaced by boreal forests, grasslands, and savannas [56]. The climate change strongly affected the vegetation cover, particularly in middle latitudes. During the middle Miocene to late Pliocene, the topography of China forms a three-step staircase in which the Yunnan–Guizhou Plateau constitutes the southern part of the second step. Since the Middle Pleistocene, the continuous uplift of the Qinghai-Tibet Plateau has promoted the formation of the Yunnan-Guizhou Plateau, which greatly affected the tectonic of the Yunnan–Guizhou Plateau, forming mountains and deep valleys and rearranging major river drainages. These events in East Asia during the second half of the Miocene may have gradually created new surface conditions that were unfavorable to species adapted to tropical habitats [51].

The divergence time in six populations of *T. yunnanensis* occurred in the middle-to-late Pleistocene. The results were similar to previous studies on *Nesticella* spiders in this region. Zhang and Li [54] found that the cave groups of *Nesticella* in the Yunnan-Guizhou Plateau originated in the Miocene, and most populations of different species formed in the Pleistocene. Further research by Ballarin and Li [8] found that climate change in the Miocene caused *Nesticella* to take refuge in caves and to begin rapid differentiation in the 5.5 Ma. We suggest that *T. yunnanensis*, which is similar to the *Nesticell* in body size and habitat, may have experienced a similar evolutionary history. Because of the absence of surface populations and closest sister species in Yunnan-Guizhou Plateau, we could not determine the origin of *T. yunnanensis*. Ideally, if we can collect related species of *T. yunnanensis* on the surface, the origin could be inferred, and how the geological change impacted the organisms in the Yunnan-Guizhou Plateau can be further explored.

## 5. Conclusions

This study systematically explored the population genetics of a cave spider in Yunnan-Guizhou Plateau. Our results suggested that the isolation was a pivotal factor increasing biodiversity of cave faunas. We believe that cave faunas with similar body sizes and habitats also have similar genetic structures in the Yunnan-Guizhou Plateau. A correct understanding of biodiversity is fundamental to conservation, and karst areas need to be further studied, because there are thousands of caves which are unexplored. Our study provides new insights into the diversity of subsurface life in karst areas. Further research could use larger datasets, such as NGS data, and new analytical tools to explore genetic structures between and even within populations. More research on the underground fauna in karst areas will shed light on the formation pattern of biodiversity in this region.

## Figures and Tables

**Table 1:** Information of spider samples and localities.

Species	Sites (Abbrs.)	Sample Size	Geographic Coordinates	Collection Localities
*Trogloneta yunnanensis*	Guanniu Cave (GN)	6♂8♀	27.6137° N, 106.9691° E	Guizhou: Zunyi City, Shenxi Twon, Longjiang Vill.
	Yelaoda Cave (YLD)	6♂6♀	27.1843° N, 105.4657° E	Guizhou: Dafang Co., Wenge Town, Sanhe Vill.
	Qingxu Cave (QX)	4♂7♀	27.1030° N, 105.6699° E	Guizhou: Dafang Co., Yangchang Town, Longdong Vill.
	Shilida Cave (SLD)	6♂7♀	25.6237° N, 104.7566° E	Guizhou: Panxian Co., Zhudong Town, Shiliping Vill.
	Xianren Cave (XR)	7♂7♀	25.4648° N, 102.1729° E	Yunnan: Wuding Co., Maojie Town
	Baiyan Cave (BY)	5♂5♀	25.1510° N, 103.4010° E	Yunnan: Yiliang Co., Jiuxiang Town, Dazhezong Vill.
*Trogloneta yuensis*	Yuelu Mt. Parkland	1♂1♀	28.1869° N, 112.9421° E	Hunan: Changsha City, Yuelu Dist.

**Table 2:** Genetic diversity indices of *Trogloneta yunnanensis*.

Populations	*cox1*				*16S*				*H3*				*ITS-2*			
	N	H	π	Hd	N	H	π	Hd	N	H	π	Hd	N	H	π	Hd
XR	13	1	0.00000	0.000	13	2	0.00336	0.282	13	1	0.00000	0.000	13	1	0.00000	0.000
QX	11	2	0.00052	0.327	11	2	0.00043	0.182	11	2	0.00175	0.545	11	1	0.00000	0.000
SLD	13	3	0.00098	0.564	13	2	0.00366	0.385	13	2	0.00090	0.282	13	1	0.00000	0.000
YLD	12	2	0.00026	0.167	12	2	0.00040	0.167	12	1	0.00000	0.000	12	2	0.00082	0.303
GN	13	1	0.00000	0.000	14	1	0	0	14	2	0.00116	0.363	13	1	0.00000	0.000
BY	10	3	0.00138	0.511	10	3	0.00450	0.511	10	1	0.00000	0.000	10	1	0.00000	0.000

Notes: N, number of individuals; H, number of haplotypes; π, nucleotide diversity; h, haplotype diversity.

**Table 3:** Pairwise *F*_ST_ among populations based on *cox1*.

	XR	QX	SLD	YLD	GN
QX	0.99 ***				
SLD	0.98 ***	0.96 ***			
YLD	0.99 ***	0.97 ***	0.97 ***		
GN	1.00 ***	0.99 ***	0.98 ***	0.97 ***	
BY	0.97 ***	0.96 ***	0.92 ***	0.97 ***	0.98 ***

***: *p* < 0.01.

**Table 4:** Gene flow among populations based on *cox1*.

	XR	QX	SLD	YLD	GN
QX	0.01				
SLD	0.01	0.02			
YLD	0.01	0.02	0.02		
GN	0.00	0.01	0.01	0.02	
BY	0.02	0.02	0.04	0.02	0.01

**Table 5:** Mean pairwise uncorrected *p*-distances among populations based on *cox1*.

	XR	QX	SLD	YLD	GN
QX	0.0184				
SLD	0.0253	0.0210			
YLD	0.0109	0.0142	0.0212		
GN	0.0151	0.0178	0.0253	0.0049	
BY	0.0222	0.0209	0.0149	0.0210	0.0252

**Table 6:** AMOVA analysis based on *cox1*.

Source of Variation	d. f.	Sum of Sequence	Variance Components	Percentage of Variation
Among populations	5	346.132	5.767	97.4
Within populations	66	10.145	0.153	2.6
Total	71	356.278	5.920	100

## Data Availability

The gene sequences generated in this study have been deposited in GenBank with accession numbers listed in Table S2.

## Supplementary Materials

The following supporting information can be downloaded at: https://www.mdpi.com/article/10.3390/ani13071244/s1, Figure S1: Concatenated gene tree constructed in IQ-TREE; Table S1: Primers used in this study; Table S2: Sequences information; Table S3: Best-fit models for genes. References [57,58,59,60,61,62] are cited in the supplementary materials.
