# Supplementary material for: New Insights into the Variation and Admixture of the Cave-Dwelling Spider Trogloneta yunnanensis in South China Karst
Source: Animals (Basel). 2023 Apr 3;13(7):1244. doi: 10.3390/ani13071244 (PMC10093053; doi:10.3390/ani13071244)
Supplement: Supplementary file 1 [file animals-13-01244-s001.zip › animals-2255470-supplementary.pdf]

**Table S1.** Primers used in this study

| Locus | | Primer | Sequence 5'→3'<br>（forward and reverse） | Reference | PCR reaction |
|---|---|---|---|---|---|
| ITS-2 | F | ITS-5.8s | GGGACGATGAAGAACGGAGC | (White et al. 1990) | 94°C 45s<br>48°C 1min<br>72°C 60s |
| | R | ITS-28s | TCCTCCGCTTATTGATATGC | | |
| 28SD2 | F | 28sa | GACCCGTCTTGAAACACGGA | (Rix et al. 2008) | 94°C 30s<br>55°C 30s<br>72°C 30s |
| | R | LSUR | GCTACTACCACCAAGATCTGCA | | |
| H3 | F | H3nf | ATGGCTCGTACCAAGCAGAC | (Colgan et al. 1998) | 94°C 30s<br>50°C 45s<br>72°C 45s |
| | R | H3nr | ATRTCCTTGGGCATGATTGTTAC | | |
| cox1 | F | LCO1490 | GGTCAACAAATCATAAAGATATTGG | (Folmer et al. 1994) | 94°C 30s<br>49°C 30s<br>72°C 30s |
| | R | HCO2198 | TAAACTTCAGGGTGACCAAAAAATCA | | |
| 16S | F | 16sb2_12864 | CTCCGGTTTGAACTCAGATCA | (Hormiga et al. 2003) | 94°C 30s<br>50°C 30s<br>72°C 45s |
| | R | LR-J-13360 | GTAAGGCCTGCTCAATGA | (Yan 2018) | |

**Table S2.** Sequences information

| Samples | | GenBank accession number | | | | |
|---|---|---|---|---|---|---|
| Species | individual code | *cox1* | 16S | ITS-2 | 28S | H3 |
| *Trogloneta yunnanense* | GN-1 | MZ577427 | MZ612904 | OK513497 | MZ613029 | MZ603566 |
| | GN-2 | NA | MZ612905 | NA | MZ613030 | MZ603567 |
| | GN-3 | MZ577428 | MZ612906 | OK513498 | MZ613031 | MZ603568 |
| | GN-4 | MZ577429 | MZ612907 | OK513499 | MZ613032 | MZ603569 |
| | GN-5 | MZ577430 | MZ612908 | OK513500 | MZ613033 | MZ603570 |
| | GN-6 | MZ577431 | MZ612909 | OK513501 | MZ613034 | MZ603571 |
| | GN-7 | MZ577432 | MZ612910 | OK513502 | MZ613035 | MZ603572 |
| | GN-8 | MZ577433 | MZ612911 | OK513503 | MZ613057 | MZ603595 |
| | GN-9 | MZ577434 | MZ612912 | OK513504 | MZ613063 | MZ603601 |
| | GN-10 | MZ577435 | MZ612913 | OK513505 | MZ613058 | MZ603596 |
| | GN-11 | MZ577436 | MZ612914 | OK513506 | MZ613059 | MZ603597 |
| | GN-12 | MZ577437 | MZ612915 | OK513507 | MZ613060 | MZ603598 |
| | GN-13 | MZ577438 | MZ612916 | OK513508 | MZ613061 | MZ603599 |
| | GN-14 | MZ577439 | MZ612917 | OK513509 | MZ613062 | MZ603600 |
| | YLD-1 | MZ577392 | MZ612869 | OK513485 | MZ613024 | MZ603560 |

| | | | | | | |
|---|---|---|---|---|---|---|
| | YLD-2 | MZ577393 | MZ612870 | OK513486 | MZ613025 | MZ603561 |
| | YLD-3 | MZ577394 | MZ612871 | OK513487 | MZ613026 | MZ603562 |
| | YLD-4 | MZ577395 | MZ612872 | OK513488 | MZ613027 | MZ603563 |
| | YLD-5 | MZ577396 | MZ612873 | OK513489 | MZ613028 | MZ603564 |
| | YLD-6 | MZ577397 | MZ612874 | OK513490 | MZ613024 | MZ603565 |
| | YLD-7 | MZ577398 | MZ612875 | OK513491 | MZ613051 | MZ603589 |
| | YLD-8 | MZ577399 | MZ612876 | OK513492 | MZ613052 | MZ603590 |
| | YLD-9 | MZ577400 | MZ612877 | OK513493 | MZ613053 | MZ603591 |
| | YLD-10 | MZ577401 | MZ612878 | OK513494 | MZ613054 | MZ603592 |
| | YLD-11 | MZ577402 | MZ612879 | OK513495 | MZ613055 | MZ603593 |
| | YLD-12 | MZ577403 | MZ612880 | OK513496 | MZ613056 | MZ603594 |
| | QX-1 | MZ577440 | MZ612918 | OK513461 | MZ613012 | MZ603548 |
| | QX-2 | MZ577441 | MZ612919 | OK513462 | MZ613013 | MZ603549 |
| | QX-3 | MZ577442 | MZ612920 | OK513463 | MZ613014 | MZ603550 |
| | QX-4 | MZ577443 | MZ612921 | OK513464 | MZ613015 | MZ603551 |
| | QX-5 | MZ577444 | MZ612922 | OK513465 | MZ613016 | MZ603552 |
| | QX-6 | MZ577445 | MZ612923 | OK513466 | MZ613045 | MZ603583 |
| | QX-7 | MZ577446 | MZ612924 | OK513467 | MZ613046 | MZ603584 |
| | QX-8 | MZ577447 | MZ612925 | OK513468 | MZ613047 | MZ603585 |
| | QX-9 | MZ577448 | MZ612926 | OK513469 | MZ613048 | MZ603586 |
| | QX-10 | MZ577449 | MZ612927 | OK513470 | MZ613049 | MZ603587 |
| | QX-11 | MZ577450 | MZ612928 | OK513471 | MZ613050 | MZ603588 |
| | SLD-1 | MZ577404 | MZ612881 | OK513472 | MZ613017 | MZ603553 |
| | SLD-2 | MZ577405 | MZ612882 | OK513473 | MZ613018 | MZ603554 |
| | SLD-3 | MZ577406 | MZ612883 | OK513474 | MZ613019 | MZ603555 |
| | SLD-4 | MZ577407 | MZ612884 | OK513475 | MZ613020 | MZ603556 |
| | SLD-5 | MZ577408 | MZ612885 | OK513476 | MZ613021 | MZ603557 |
| | SLD-6 | MZ577409 | MZ612886 | OK513477 | MZ613022 | MZ603558 |
| | SLD-7 | MZ577410 | MZ612887 | OK513478 | MZ613023 | MZ603559 |
| | SLD-8 | MZ577411 | MZ612888 | OK513479 | MZ613039 | MZ603577 |
| | SLD-9 | MZ577412 | MZ612889 | OK513480 | MZ613040 | MZ603578 |
| | SLD-10 | MZ577413 | MZ612890 | OK513481 | MZ613041 | MZ603579 |
| | SLD-11 | MZ577414 | MZ612891 | OK513482 | MZ613042 | MZ603580 |
| | SLD-12 | MZ577415 | MZ612892 | OK513483 | MZ613043 | MZ603581 |
| | SLD-13 | MZ577416 | MZ612893 | OK513484 | MZ613044 | MZ603582 |
| | XR-1 | MZ577379 | MZ612856 | OK513448 | MZ613005 | MZ603541 |
| | XR-2 | MZ577380 | MZ612857 | OK513449 | MZ613006 | MZ603542 |
| | XR-3 | MZ577381 | MZ612858 | OK513450 | MZ613007 | MZ603543 |
| | XR-4 | MZ577382 | MZ612859 | OK513451 | MZ613008 | MZ603544 |
| | XR-5 | MZ577383 | MZ612860 | OK513452 | MZ613009 | MZ603545 |
| | XR-6 | MZ577384 | MZ612861 | OK513453 | MZ613010 | MZ603546 |
| | XR-7 | MZ577385 | MZ612862 | OK513454 | MZ613011 | MZ603547 |

| | | | | | | |
|---|---|---|---|---|---|---|
| | XR-8 | MZ577386 | MZ612863 | OK513455 | MZ613065 | MZ603603 |
| | XR-9 | MZ577387 | MZ612864 | OK513456 | MZ613066 | MZ603604 |
| | XR-10 | MZ577388 | MZ612865 | OK513457 | MZ613067 | MZ603605 |
| | XR-11 | MZ577389 | MZ612866 | OK513458 | MZ613068 | MZ603606 |
| | XR-12 | MZ577390 | MZ612867 | OK513459 | MZ613069 | MZ603607 |
| | XR-13 | MZ577391 | MZ612868 | OK513460 | MZ613064 | MZ603602 |
| | BY-1 | MZ577417 | MZ612894 | OK513510 | MZ613036 | MZ603573 |
| | BY-2 | MZ577418 | MZ612895 | OK513511 | NA | MZ603574 |
| | BY-3 | MZ577419 | MZ612896 | OK513512 | MZ613037 | MZ603575 |
| | BY-4 | MZ577420 | MZ612897 | OK513513 | MZ613038 | MZ603576 |
| | BY-5 | MZ577421 | MZ612898 | OK513514 | MZ613070 | MZ603608 |
| | BY-6 | MZ577422 | MZ612899 | OK513515 | MZ613071 | MZ603609 |
| | BY-7 | MZ577423 | MZ612900 | OK513516 | MZ613072 | MZ603610 |
| | BY-8 | MZ577424 | MZ612901 | OK513517 | MZ613073 | MZ603611 |
| | BY-9 | MZ577425 | MZ612902 | OK513518 | MZ613074 | MZ603612 |
| | BY-10 | MZ577426 | MZ612903 | OK513519 | MZ613075 | MZ603613 |
| *Trogloneta yuensis* | XX52f | MZ584802 | MZ612929 | NA | MZ613076 | MZ603614 |
| | XX52m | MZ584803 | MZ612930 | NA | MZ613077 | MZ603615 |

**Table S3.** Best-fit models for genes

| locus | Mrbayes | BEAST | IQTREE |
|---|---|---|---|
| *cox1* | GTR+I+G | HKY+I+G | TIM3 + F + I + G4 |
| 16S | GTR+I+G | HKY+I+G | TIM3 + F + I + G4 |
| H3 | GTR+G | HKY+I | TIM + F + G4 |
| 28S | HKY+G | HKY+I | HKY + F |
| ITS-2 | GTR+G | HKY+I | TIM + F + G4 |

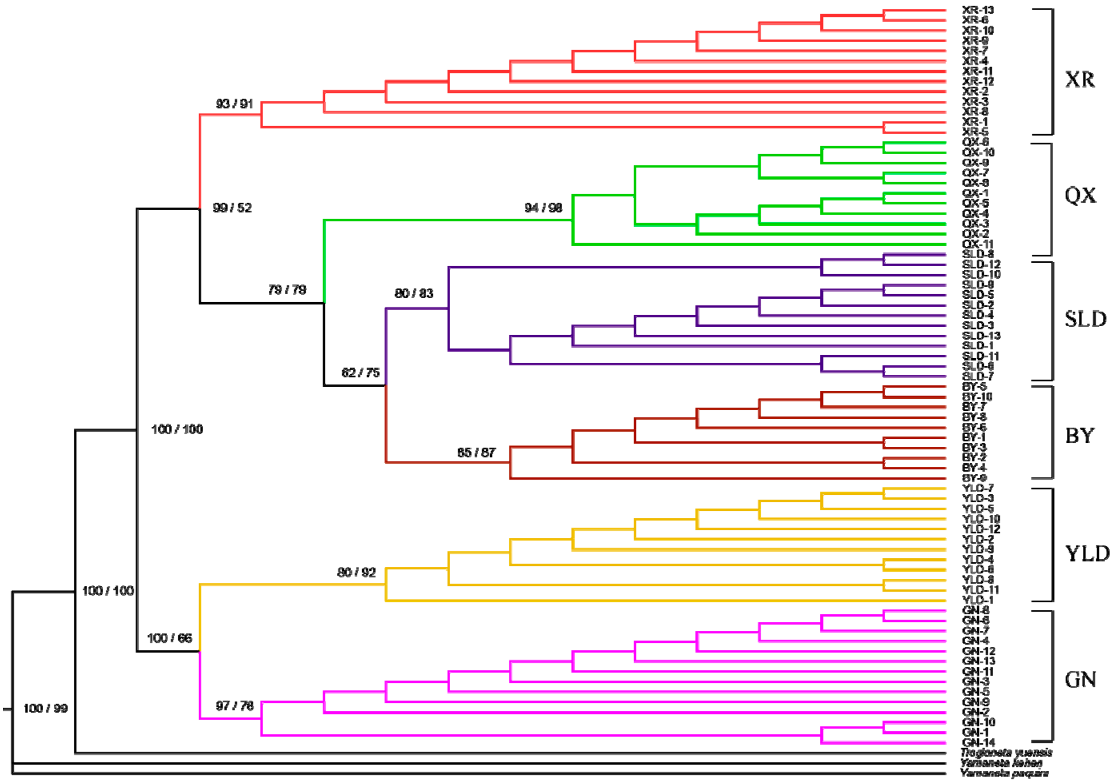


Figure S1. Concatenated gene tree constructed in IQ-TREE. Colors of branches were same as BI tree. Bootstrap supports (right) and SH-aLRT (left) values were provide beside nodes.

## Reference

Colgan DJ, McLauchlan A, Wilson GDF, Livingston SP, Edgecombe GD, Macaranas J, Cassis G, Gray MR. 1998. Histone H3 and U2 snRNA DNA sequences and arthropod molecular evolution. Australian Journal of Zoology, 46(5): 419.

Folmer O, Black M, Hoeh W, Lutz R, Vrijenhoek R. 1994. DNA primers for amplification of mitochondrial cytochrome C oxidase subunit I from diverse metazoan invertebrates. Molecular Marine Biology and Biotechnology, 3(5): 294–299.

Hormiga G, Arnedo M, Gillespie RG. 2003. Speciation on a Conveyor Belt: Sequential Colonization of the Hawaiian Islands by Orsonwelles Spiders (Araneae, Linyphiidae). Systematic Biology, 52(1): 70–88.

Rix MG, Harvey MS, Roberts JD. 2008. Molecular phylogenetics of the spider family Micropholcommatidae (Arachnida: Araneae) using nuclear rRNA genes (18S and 28S). Molecular phylogenetics and evolution, **46**, 1031–1048.

White TJ, Bruns T, Lee S, Taylor J. 1990. Amplification and direct sequencing of fungal ribosomal RNA genes for phylogenetics. In: PCR Protocols: a guide to methods and applications. New York: Academic Press.

Yan FH. 2018. Molecular systematics of the Mysmenidae (Arachnida, Araneae) from China. M. S. Thesis, Sichuan University, Chengdu.
